# Dynamic DNA Nanomachines for Biosensing and Drug Delivery

**DOI:** 10.3390/s26113411

**Published:** 2026-05-28

**Authors:** Borui Zhang, Mengyao Sun, Jie Chao

**Affiliations:** 1 State Key Laboratory for Flexible Electronics (LoFE), Nanjing University of Posts and Telecommunications, Nanjing 210023, China; 2 Jiangsu Key Laboratory of Smart Biomaterials and Theranostic Technology, Nanjing University of Posts and Telecommunications, Nanjing 210023, China; 3 College of Materials Science and Engineering, Nanjing University of Posts and Telecommunications, Nanjing 210023, China; 4 Key Laboratory of Drug Quality Control and Pharmacovigilance Ministry of Education, China Pharmaceutical University, Nanjing 210009, China

**Keywords:** DNA nanostructures, dynamic nanomachines, stimulus-response strategies, biosensing, drug delivery

## Abstract

DNA nanotechnology exploits the high precision and programmability of base complementary pairing to construct diverse nanostructures. Especially, the integration of stimuli-responsive modules has driven the evolution of DNA nanostructures toward dynamic nanomachines, conferring significant advantages in biomedical applications. In this review, the stimulus–response strategies of DNA nanostructures are first outlined, including molecular-driven mechanisms and environmental stimulation methods. The recent advances in the application of nanomachines based on various response strategies in biosensing and drug delivery are then elaborated. Finally, current challenges and prospects for these nanomachines in clinical diagnosis and precision therapy are discussed. This review provides a systematic reference for the development of responsive DNA nanomachines and the exploration of their biomedical applications.

## 1. Introduction

DNA nanotechnology, with its capabilities in programmable self-assembly and nanoscale precision, has emerged as a pivotal platform for the design of functional nanomaterials [1]. Based on the precise programmability of base pairing, DNA can self-assemble into complex structures, including one-dimensional nanowires, two-dimensional lattices, and three-dimensional frameworks [2,3,4,5]. By virtue of its spatially addressable nature and ease of chemical modification, DNA nanostructures have significantly advanced the rapid development of molecularly precise assembly and biointerface engineering. Early efforts in DNA nanotechnology primarily focused on enhancing structural scalability and geometric complexity [6,7,8,9,10,11]. However, conventional DNA nanostructures remain limited in their ability to address dynamic changes in complex biological environments and to achieve precise spatiotemporally controlled interventions [12,13].

Inspired by the sophisticated functions of biological molecular machines, endowing nanostructures with dynamic motion capabilities has become a research hotspot aimed at further advancing the rational design of DNA nanotechnology [14,15,16]. DNA nanostructures are increasingly evolving into an ideal platform that integrates complex actuating components and multifunctional modules. The development and integration of stimulus-responsive modules, which drive the transition of DNA nanostructures from static architectures to dynamic nanomachines, have emerged as a central driving force in the field [17]. Such dynamic nanomachines can sensitively respond to specific molecular signals or external environmental stimuli, enabling precise functional regulation through conformational rearrangements, structural dissociation, or actuation behaviors [18,19,20,21]. Collectively, these advantages endow dynamic DNA nanomachines with significant potential for biomedical applications.

**Scheme 1:**
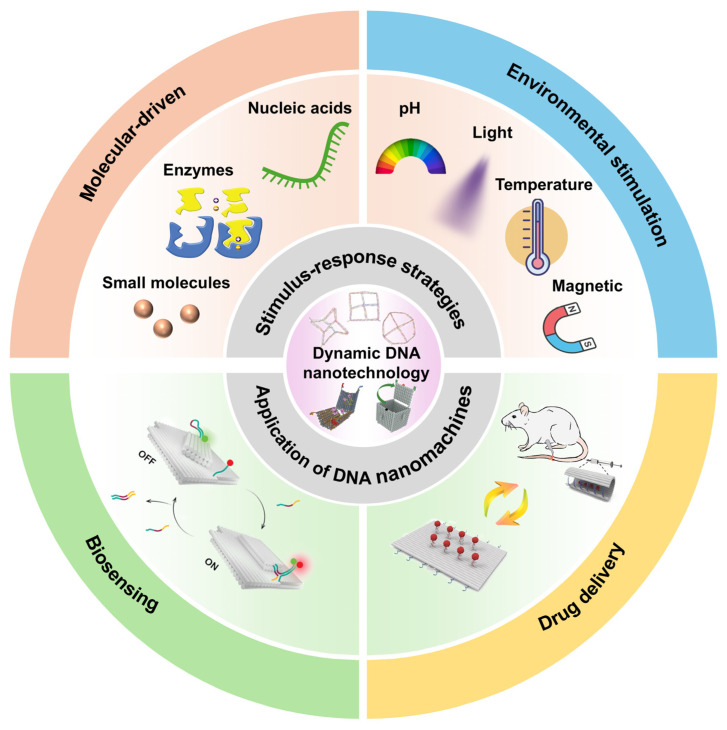
Schematic overview of the stimulus-responsive strategies of dynamic DNA nanomachines and their biomedical applications.

Although several existing reviews have briefly summarized the progress of DNA nanotechnology and its biomedical applications. However, the mechanistic relationship between stimulus-responsive structural reconfiguration and functional biomedical outputs has not been comprehensively summarized. This review systematically surveys the emerging field of dynamic DNA nanomachines for biomedical applications (Scheme 1). We first highlight the stimulus–response strategies of DNA nanomachines, including molecular-driven mechanisms and environmental stimulation methods. We further outline the translation of these programmable dynamic behaviors into advanced biomedical functions, particularly in biosensing and controlled drug delivery. Finally, we discuss the current challenges and prospects of these nanomachines in clinical diagnosis and precise treatment. The review aims to provide critical insights to advance the intelligent development of dynamic DNA nanomachines, thereby addressing key challenges in clinical implementation.

## 2. Stimulus-Responsive Dynamic DNA Nanomachines

The design of DNA nanomachines typically begins with the hierarchical assembly of structurally distinct modules. In these nanomachines, rigid domains provide structural stability, flexible hinges and compliant joints enable local motion, and stress-tunable connecting elements facilitate conformational transitions between predefined metastable states. Several seminal studies have systematically elucidated these foundational design principles, establishing both the theoretical and practical framework for programmable DNA nanomachines. For instance, Castro et al. demonstrated how the strategic combination of rigid and flexible domains can control DNA origami mechanics and global shape reconfiguration [22]. Dietz et al. introduced rigid-body design concepts and energy-based modeling to predict and program nanoscale motion in DNA origami systems [23].

By rationally arranging DNA mechanical components, DNA nanomachines can exhibit coordinated deformations in response to specific stimuli [20,24]. The dynamic behavior of DNA nanomachines arises from the programmable architecture of their structures and the way energy landscapes govern conformational transitions. Local interactions and geometric stability determine the relative stability of alternative conformational states, allowing structural transitions to be reversibly controlled. Gerling et al. demonstrated reconfigurable DNA devices and assemblies formed by shape-complementary 3D components, showing that structural motion can be reversibly controlled by the balance between attractive and repulsive interactions, thereby regulating the conformations of the assemblies [25]. This highlights how energy minima and barriers can be designed to achieve precise, reversible structural changes. Madhvacharyula et al. realized mechanical frustration in a DNA origami Kagome lattice. In this system, selective actuation of reconfigurable struts drives the structure into one of two states: a homogeneous, strain-distributing state or a strain-localizing frustrated state. Each state corresponds to a distinct global free-energy landscape and a switchable overall conformation [26].

Furthermore, the local sequence inputs are converted into global mechanical responses, enabling increasingly sophisticated nanoscale actuation behaviors. For example, Song et al. developed DNA relay arrays assembled from dynamic antijunction units, where a small amount of trigger can induce global deformation in a domino-like manner [27]. Ji et al. developed a topology-programmed DNA origami system that undergoes global conformational changes through glue-cut operations, enabling the structural transformation from a 2D planar sheet into a 3D interconnected architecture and subsequently back into a 2D frame. This process dynamically reconfigures the network topology and enables multipurpose logic computation [28].

On this basis, different types of stimuli drive these nanostructures to perform functional motions through specific mechanisms. Based on the features of the triggering signal, current responsive strategies of dynamic DNA nanomachines can generally be classified into two categories: molecular-driven mechanisms and environmental stimulation methods. Molecular-driven mechanisms mainly rely on specific molecular recognition events, such as sequence complementarity, aptamer-target binding, or enzyme-substrate interactions, which locally regulate strand hybridization, displacement, or catalytic processing. In contrast, environmental stimulation methods are generally governed by global physicochemical changes in the surrounding environment, including variations in pH, ion concentration, temperature, light, or external physical fields, which collectively alter the thermodynamic stability or mechanical state of DNA nanostructures. The following sections summarize the fundamental mechanisms, representative examples, and defining features of these two strategies.

### 2.1. Molecular-Driven Mechanisms

#### 2.1.1. Nucleic Acids

**Figure 1:**
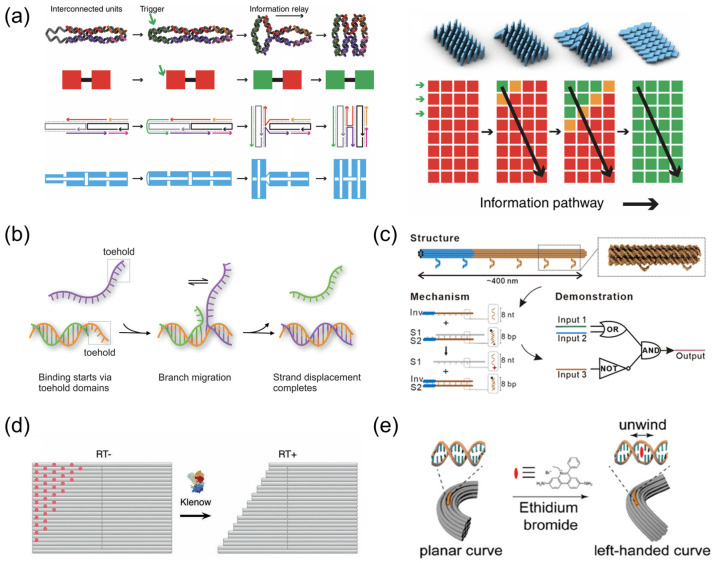
Molecular-driven mechanisms. (**a**) DNA relay arrays assembled from dynamic antijunction units. Conformational information is relayed from the converted unit to its nearest neighbors. The green arrow indicates the triggered signal. Stable conformations “Red” and “Green”, and unstable conformation “Orange”. Reprinted with permission from ref. [27]. (**b**) Toehold-mediated strand displacement (TMSD) mechanism. Reprinted with permission from ref. [34]. (**c**) Meta-toehold-mediated meta-DNA strand displacement strategy. Reprinted with permission from ref. [37]. (**d**) Site-specific cutting of rectangular DNA origami by polymerase-triggered strand displacement. Reprinted with permission from ref. [45]. (**e**) Ethidium bromide intercalation induces local unwinding of double-stranded DNA and global conformational rearrangement. Reprinted with permission from ref. [52].

Nucleic acid strands serve as the most employed molecular triggers for driving DNA nanomachines to achieve controllable motion or structural transformation [20,29]. In some systems, nucleic acid strands can directly act as signals for structural reconfiguration by pairing with predefined regions and thereby reshaping the overall architecture. For example, Song et al. developed DNA relay arrays assembled from dynamic antijunction units, in which an input DNA strand first binds to a selected unit and switches its local conformation. This local alteration modifies the interface with the neighboring unit and destabilizes the original base-stacking pattern, thereby inducing the adjacent unit to adopt the same conformation. Consequently, a small number of input strands can trigger a domino-like relay of structural change across the entire array, culminating in long-range reconfiguration [27] (Figure 1a). Building on this concept, a growing number of related systems have been developed. A similar direct triggering mechanism was later applied in modular reconfigurable DNA origami, where trigger strands switch individual anti-junction modules between two local conformations. These module-level shape changes are then propagated through the connected structure, ultimately enabling higher-order reconfiguration such as the conversion of 2D sheets into 3D architectures [30]. Fan et al. further developed reconfigurable DNA origami domino arrays (DODAs) and further applied them to molecular information encryption. In this system, nucleic acid strands induced conformational reconfiguration of the DNA nanostructure, and the resulting structural change served as the key for encoding and decoding hidden information [31,32]. Cole et al. further developed a DNA origami array in which trigger DNA strands directly hybridized to edge anti-junctions and initiated a stepwise allosteric conformational relay. Using this mechanism, they further demonstrated the release of a cargo DNA strand at a predefined stage of the reaction cascade, highlighting the potential of this system for tunable mechanochemical coupling with spatial and temporal control [33].

Moreover, the various reported nucleic acid-driven nanostructures typically rely on the toehold-mediated strand displacement reaction (TMSD) [34] (Figure 1b). In this process, an invading strand first binds to an exposed toehold and then displaces the incumbent strand through branch migration, ultimately forming a new duplex. The high programmability of the toehold region allows its sequence to be arbitrarily designed, enabling it to function in a highly independent manner within the structure and permitting DNA nanomachines to generate specific motions or conformational transformations, including opening and closing, bending, rotation, and displacement [24,35]. For example, Thubagere et al. constructed a cargo-sorting DNA robot in which strand displacement served as the core mechanism for multiple functions. Reversible strand displacement between adjacent track strands enabled random walking, whereas irreversible strand displacement allowed cargo pickup and cargo drop-off at designated goals. In this way, different mechanical tasks were integrated into one system through modular strand-displacement reactions, enabling autonomous cargo sorting on a DNA origami surface [36]. Qi et al. further extended strand displacement to submicron-scale DNA architectures by developing a meta-toehold-mediated meta-DNA strand displacement reaction. The invader first binds to the meta-toehold of the substrate, forms a triple-stranded intermediate, and then displaces the original strand through branch-migration-like exchange of meta-bases, thereby generalizing the core mechanism of TMSD from conventional DNA strands to hierarchical meta-DNA systems [37] (Figure 1c).

Beyond the classical TMSD technique, further research on strand displacement itself has significantly expanded the design space of nucleic acid-driven molecular mechanisms. Specifically, several noncanonical strand displacement modes and regulatory strategies have been developed to overcome the limitations of conventional TMSD, such as its susceptibility to nonspecific interactions, crosstalk, and interference in complex biological environments [38,39,40]. For example, Liu et al. proposed a toehold-independent strand displacement reaction network. In this network, the gain in configurational entropy arising from the release of multiple strands serves as the driving force, and this principle was further extended to cascade signal transmission and DNA logic circuits [41]. Talbot et al. introduced a mismatch-induced, toehold-free strand displacement strategy, in which mismatched base pairs destabilize the initial complex and thereby promote strand exchange [42]. Similarly, Madhanagopal et al. demonstrated toehold-less strand displacement in a switchback DNA system, showing that structural reconfiguration can still proceed in the absence of an explicit toehold [43]. Weng et al. further developed a cooperative branch migration mechanism, in which complementarity within the branch migration region is used to regulate strand displacement kinetics, reset behavior, and selective activation [44].

Nucleic acid-driven triggers are highly programmable and modular, enabling precise control over complex structural reconfigurations. They allow integration of multiple mechanical tasks and high spatiotemporal precision. However, these systems can be susceptible to nonspecific interactions and crosstalk in complex environments. Additionally, increasing the number of strands or layers adds structural and synthesis complexity, which may limit in vivo applicability.

#### 2.1.2. Enzymes

In addition to nucleic acid molecules, molecular tool enzymes serve as crucial elements for achieving the controllable reconstitution of complex DNA nanostructures. Polymerases, ligases, and restriction enzymes, by virtue of their diverse DNA manipulation capabilities, can specifically interact with nanostructure components and drive structural transformation, assembly, or reconfiguration. For example, Chen et al. proposed a DNA kirigami strategy based on polymerase-triggered strand displacement, in which polymerase extension progressively removed specific staple strands. This process enabled site-specific cutting of rectangular DNA origami and programmable transformation into multiple predefined geometries [45] (Figure 1d). They subsequently introduced a “presketched DNA origami canvas” strategy, where loosely connected staple strands were preinstalled in the target cutting regions to improve the precision of polymerase-driven DNA kirigami [46]. Kang et al. later developed a synthetic molecular switch cooperatively driven by DNA polymerase and a nicking endonuclease. By controlling the exposure and sequestration of sticky ends, this system reversibly regulated DNA assembly and disassembly between “ON” and “OFF” states. It was further applied to lattice and origami reconfiguration, as well as one-step “cut-and-paste” operations, highlighting the potential of enzyme-triggered mechanisms for dynamically programming complex DNA nanostructures [47]. Bai et al. further showed that enzymatic ligation can induce reconfiguration of DNA nanostructures. By selectively ligating chosen segments, they generated local stability differences within addressable assemblies and achieved programmed structural transformation after thermal treatment [48].

Enzyme-driven mechanisms provide highly specific and programmable control over DNA nanostructures, allowing transformations such as cutting, ligation, or reconfiguration that are difficult to achieve with passive molecular triggers. They enable complex operations with high selectivity. Nevertheless, enzyme activity is sensitive to environmental conditions such as pH and temperature, and the integration of enzymes increases overall system complexity.

#### 2.1.3. Small Molecules

Small molecules can also serve as effective trigger signals. In these systems, molecular inputs may induce dynamic responses through several distinct routes, including specific molecular recognition, chemical reactivity, and physicochemical interactions.

##### Target-Aptamer Binding

In the most common design, the target molecule first binds to a recognition element, and this interaction is subsequently translated into a structural switch or conformational change. For example, Huang et al. constructed an adenosine triphosphate (ATP) and adenosine-responsive chiral plasmonic DNA origami nanomachine, in which duplex locks and split-aptamer locks acted as switchable structural constraints. Upon target binding, aptamer formation reorganized the locking state of the origami, thereby driving the nanomachine to switch between the chiral and relaxed conformations and generating a corresponding chiroptical response [49]. Similarly, Gao et al. introduced an ATP-binding aptamer into the capping module of a DNA nanotubule, thereby establishing an “ATP recognition–decapping–channel activation” response process [50]. Walter et al. further integrated a split adenosine aptamer sensor into a DNA origami platform to construct a “DNA origami traffic lights” device, in which the presence of adenosine or ATP induced aptamer assembly, triggered structural state transitions, and produced a two-color fluorescence readout [51].

##### Physical Interaction

In addition, small-molecule-triggered regulation can also arise from physical interactions. Xie et al. used ethidium bromide (EB) intercalation to induce local unwinding of double-stranded DNA and global conformational rearrangement, constructing a chemo-mechanically coupled DNA origami nanoclamp capable of generating compressive forces of up to 11.2 pN [52] (Figure 1e). Xu et al. further showed that small-molecule DNA binders can regulate dynamic DNA reactions through physicochemical interactions with double-stranded DNA. In their binder-induced nucleic acid strand displacement system, strand displacement pathways were quantitatively tuned by the affinity, charge, and concentration of the binders, thereby broadening small molecule-triggered regulation beyond aptamer-mediated recognition [53].

##### Chemical Reaction

For chemical reaction, a typical example is the regulation of DNA nanomachines through chemically cleavable dynamic covalent linkages. In these systems, endogenous reductive molecules, particularly glutathione (GSH), act as molecular triggers that induce bond cleavage and thereby drive structural transformation or cargo release. Because intracellular GSH concentrations are significantly elevated in many tumor cells, GSH-responsive dynamic covalent bonds have been widely employed in responsive nanocarrier systems and biomedical nanodevices [54]. Among these responsive motifs, disulfide bonds are the most commonly used because they exhibit good biocompatibility and moderate reduction sensitivity. For example, Wang et al. introduced disulfide-containing lock strands into a tubular DNA origami nanodevice to enable redox-responsive structural regulation. In the presence of GSH, cleavage of the disulfide bonds removed the locking elements and converted the carrier from a closed to an open state [55]. Beyond disulfide chemistry, diselenide bonds possess lower bond dissociation energies and therefore exhibit higher redox sensitivity and faster cleavage kinetics under reductive conditions [56,57]. Ditelluride bonds further enhance this responsiveness because of their even weaker bond strength, enabling ultrasensitive structural switching in response to redox-active small molecules [58].

Overall, small-molecule-responsive DNA nanomachines offer several important advantages, including high physiological relevance, autonomous activation, and compatibility with complex in vivo environments. By exploiting endogenous biomarkers such as ATP and GSH, these systems can achieve localized and controllable structural reconfiguration without relying on external instruments. Although challenges such as limited trigger specificity and potential signal interference in heterogeneous biological environments remain, their relatively simple design, favorable biocompatibility, and strong compatibility with pathological microenvironments make small-molecule-responsive strategies particularly promising for future clinical translation in precision diagnostics and targeted therapy.

### 2.2. Environmental Stimulation Methods

#### 2.2.1. Chemical Response

##### pH

**Figure 2:**
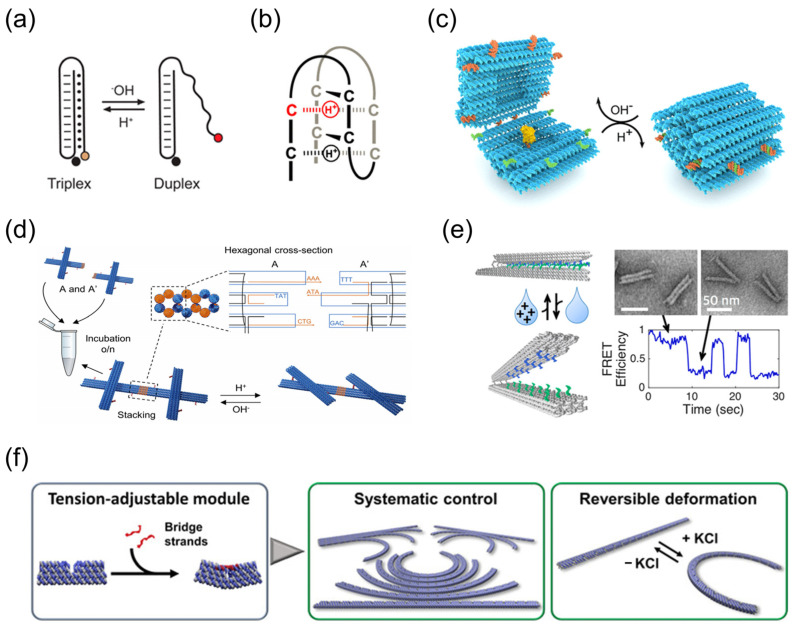
Chemical response to environmental stimulation methods. (**a**) pH-responsive triplex DNA. Reprinted with permission from ref. [59]. (**b**) pH-responsive i-motif. Reprinted with permission from ref. [61]. (**c**) DNA origami nanocapsule containing multiple programmable pH latches. Reprinted with permission from ref. [62]. (**d**) pH-driven two-dimensional lattice reconfiguration. Reprinted with permission from ref. [67]. (**e**) Cation-activated actuation method for DNA origami hinges. Reprinted with permission from ref. [70]. (**f**) DNA origami nanoarm composed of repeated tension-adjustable modules. Reprinted with permission from ref. [71].

Among environmental stimulation methods, pH stimulation is one of the most widely studied strategies because it mimics regulatory processes found in biological environments. Its basic mechanism involves altering the protonation state of nucleobases to modulate the stability of pH-responsive DNA motifs, thereby inducing conformational transitions and structural reorganization in DNA nanodevices. Two representative pH-responsive motifs are triplex DNA and the i-motif. In triplex DNA, protonation of cytosine under acidic conditions stabilizes C^+^·G-C base triplets and promotes third-strand binding, whereas increasing the pH leads to triplex dissociation [59,60] (Figure 2a). Similarly, the i-motif is formed by C-rich sequences through protonated C·C^+^ base pairing under acidic conditions and unfolds upon deprotonation at higher pH [61] (Figure 2b). These pH-responsive mechanisms have been widely used to construct dynamic DNA nanostructures with reversible switching behavior. For example, Ijäs et al. developed a DNA origami nanocapsule containing multiple programmable pH latches, in which pH-dependent formation and dissociation of triplex structures enabled reversible transitions between open and closed states [62] (Figure 2c). Lin et al. designed triplex DNA nanoswitches with distinct pH-response windows. By programming the triplex composition, they achieved pH-dependent structural transitions at different thresholds, which enabled stepwise release of reporter units under acidic and basic conditions [63]. Yang et al. further employed triplex linkers with different pH-responsive windows to realize reversible assembly/disassembly of DNA origami assemblies [64]. Majikes et al. used parallel i-motif structures to drive pH-dependent motion in DNA origami [65].

Beyond individual nanodevices, pH-responsive designs have also been extended to higher-order DNA assemblies. Wang et al. reported pH-induced symmetry conversion in DNA origami lattices, showing that pH-responsive DNA bonds can regulate lattice organization [66]. Julin et al. further extended this strategy to two-dimensional lattice reconfiguration, allowing pH changes to drive larger-scale DNA origami structures to switch between different conformations [67] (Figure 2d).

pH-responsive strategies exploit motifs such as i-motifs and triplex DNA to achieve reversible structural switching that mimics biological regulation. These systems are relatively simple to implement, highly programmable, and readily compatible with higher-order DNA assemblies. More importantly, because acidic microenvironments are widely associated with tumors, inflammation, and intracellular compartments, pH-responsive DNA nanomachines exhibit considerable potential for clinically relevant targeted drug delivery and activatable therapeutics.

##### Ions

In dynamic DNA nanomachines, ion-responsive strategies are primarily realized by converting specific ion input signals into structural conformational transitions, including ion-stabilized secondary structures and metal ion-dependent catalytic modules. A representative strategy is to use monovalent cations to regulate G-quadruplex formation. For example, Yang et al. employed G-quadruplex-forming bridges to reversibly assemble and disassemble DNA origami dimers. In this system, K^+^ induced the transition of the bridging strands from duplex-forming single strands to folded G-quadruplexes, thereby driving dimer dissociation, whereas removal of K^+^ restored the associated state [68]. This concept was later extended from discrete dimer switching to the reconfiguration of higher-order DNA nanoarchitectures. Chan et al. introduced G-quartet toeholds as adhesive units, in which Na^+^ or K^+^ stabilized G-quadruplex formation promoted polymerization of mini-square DNA building blocks, while sequential treatment with cations and chelating agents enabled reversible cycling between discrete and polymeric states [69]. In addition, Marras et al. further showed that elevated cation concentrations could drive collective hybridization of weakly complementary overhangs and thereby induce rapid, reversible opening and closing of DNA origami hinges, extending ion responsiveness from assembly control to direct conformational actuation [70] (Figure 2e). Ion-responsive modules can also induce mechanical deformation rather than simple assembly transitions. In this regard, Suzuki et al. designed a DNA origami nanoarm composed of repeated tension-adjustable modules. Here, K^+^ induced G-quadruplex formation contracted the linker strands and cumulatively transformed the structure from a linear to an arched conformation in a reversible manner [71] (Figure 2f). In addition to noncovalent structural switching, metal ions can also trigger more complex functional reconfiguration through catalytic cleavage. Wang et al. demonstrated that Zn^2+^- and Pb^2+^-dependent DNAzymes could selectively unlock “window” domains in DNA origami tiles, thereby generating nanoholes in a programmed manner and activating catalytic processes within the resulting confined nanocavities [72].

Ion-responsive modules allow rapid and reversible control over DNA nanostructures through mechanisms such as G-quadruplex formation or metal-ion catalysis. They can regulate both assembly/disassembly and local conformations, supporting dynamic actuation. However, they are sensitive to competing ions, and excessive ion concentrations can lead to unwanted aggregation or cytotoxic effects, requiring careful tuning.

#### 2.2.2. Physical Response

##### Light

**Figure 3:**
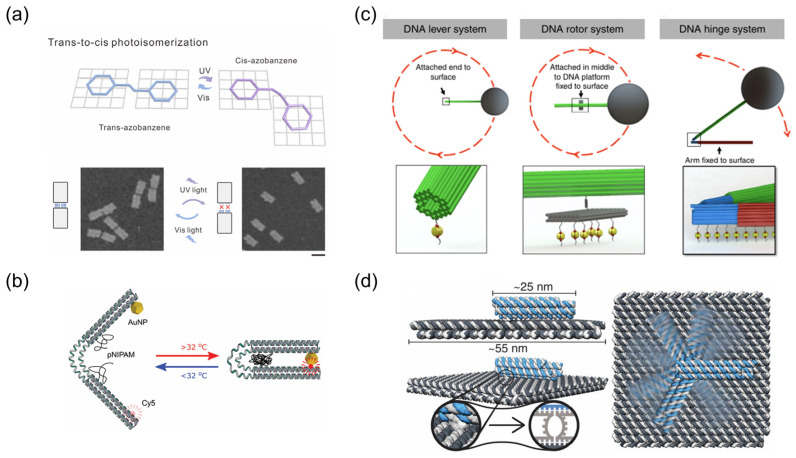
Physical response to environmental stimulation methods. (**a**) Azobenzene-mediated photocontrol of DNA origami assembly. Reprinted with permission from ref. [76]. (**b**) DNA origami flexor functionalized on both sides of the flexible hinge with poly(N-isopropylacrylamide) (PNIPAM). Reprinted with permission from ref. [80]. (**c**) Magnetically actuated DNA lever, rotor, and hinge systems integrated with micron-scale lever arms and magnetic beads. Reprinted with permission from ref. [83]. (**d**) Electric-field-actuated DNA nanorobotic arm for programmable mechanical motion. Reprinted with permission from ref. [86].

Light control technology, characterized by non-invasiveness, fast reversible response, and high spatiotemporal resolution, has become one of the ideal means for the precise manipulation of dynamic DNA nanomachines. This strategy typically relies on incorporating photoswitchable molecules, such as azobenzene, into DNA architectures [73]. Upon irradiation with ultraviolet or visible light, these molecules undergo trans-cis isomerization, which alters local hybridization stability or binding states and thereby induces conformational switching of DNA nanostructures [74]. This mechanism has been broadly applied to the design of reversible light-responsive systems. For example, Kuzyk et al. developed a reconfigurable DNA origami plasmonic template in which azobenzene isomerization at the molecular scale was converted into geometric rearrangements at the 10–100 nm scale, leading to reversible modulation of circular dichroism (CD) signals [75]. Similarly, Sun et al. incorporated azobenzene-modified linkers between multiple DNA origami modules, enabling reversible reconfiguration of multicomponent assemblies by alternately regulating the association and dissociation between DNA origami modules under ultraviolet and visible light irradiation [76] (Figure 3a). Kim et al. introduced azobenzene molecules into glue strands and crease handles of DNA wireframe origami. By switching between ultraviolet and visible light, they achieved reversible folding and unfolding of DNA origami papers, thereby extending light responsiveness from local connection switching to programmable paper-folding reconfiguration with repeatability and orthogonality [77].

Light control technology is not only used to directly regulate structural conformations but can also indirectly control the dynamic behavior of DNA nanomachines by altering the surrounding chemical environment. Ryssy et al. used a merocyanine-based photoacid to release protons under blue-light irradiation, which closed triplex DNA locks and reversibly reconfigured chiral plasmonic DNA origami assemblies, with the extent of chirality change tunable by light intensity [78]. Similarly, Berg et al. used a visible-light-responsive photoacid to drive A-motif-mediated assembly of DNA origami bricks into micron-length fibrils. Upon removal of the light stimulus, the fibrils disassembled into monomeric units [79].

Light provides non-invasive, reversible, and highly precise control over DNA nanostructures. Using photoswitchable molecules, structural changes can be remotely triggered with high spatiotemporal resolution. The limitations include shallow tissue penetration for UV/visible light and the need for chemical incorporation of photoactive groups, which may increase complexity and affect biocompatibility.

##### Temperature

The temperature-driven mechanism utilizes stimulus-induced phase transitions or local molecular interactions to regulate the dynamic transformation of DNA nanomachines. One common strategy is to couple thermoresponsive polymers with DNA nanostructures and use their phase transition behavior near the lower critical solution temperature (LCST) to induce reversible structural deformation. For example, Turek et al. functionalized both sides of the flexible hinge of a DNA origami flexor with the thermoresponsive polymer poly(N-isopropylacrylamide) (PNIPAM). As PNIPAM switched from a hydrophilic extended state to a hydrophobic collapsed state near the LCST, the origami structure underwent reversible opening and closing [80] (Figure 3b). Li et al. further developed a DNA nanomachine with both thermal and enzymatic responsiveness, in which the temperature-dependent conformational transition of PNIPAM enabled the capture and encapsulation of nucleic acid cargo, followed by its subsequent release under specific conditions [81]. Furthermore, temperature responsiveness can also arise directly from the thermal melting behavior of DNA hybridized domains. Ma et al. constructed DNA nanojoints composed of two interlocked double-stranded DNA rings, in which reversible melting and rehybridization of local duplex regions at different temperatures enabled switching between static and mobile states [82].

Temperature-responsive strategies leverage phase transitions or DNA melting to reversibly modulate structure. They are relatively straightforward in vitro and can be combined with thermoresponsive polymers. Limitations arise from the narrow physiological temperature range in vivo and the potential sensitivity of cargo or the DNA nanostructure to repeated thermal cycling.

##### Magnetic Field

Magnetic field-responsive strategies, as a non-invasive dynamic control technology, can remotely apply forces that penetrate complex media, thereby enabling reversible structural actuation of DNA nanomachines. In most reported systems, magnetic responsiveness is not achieved through intrinsic changes in DNA base pairing, but through hybrid integration of DNA nanostructures with magnetic components, thereby converting field-induced motion into nanoscale reconfiguration or function. A representative example was reported by Lauback et al., who integrated DNA origami rod, rotor, and hinge devices with stiff micron-scale lever arms and magnetic beads, enabling direct real-time actuation into many prescribed conformations with sub-second response times and operating frequencies up to several hertz [83] (Figure 3c). Lin et al. developed a magnetically powered nanomachine with a DNA clutch, in which DNA or RNA inputs reversibly engaged or disengaged force transmission between a magnetic core and a porous spherical rotor, thereby coupling molecular recognition with magnetically powered mechanical output [84]. Wu et al. further applied magnetic actuation to a probe of a DNA tetrahedral-modified nanomachine. In this system, field-driven motion promoted probe transport and target capture, thereby improving the efficiency of intracellular bioanalysis. This example further illustrates that magnetic fields can be used not only for structural actuation, but also for functional enhancement of DNA-based nanomachines in complex environments [85].

Magnetic actuation enables remote, reversible control and can penetrate complex media, making it suitable for mechanical actuation at a distance. However, it requires integration of magnetic components, which increases system size and complexity, and fine control at the nanoscale can be challenging.

##### Electric Field

Electric fields also possess non-invasive characteristics, thereby avoiding the introduction of byproducts into the system. Through external regulation, electric fields can drive DNA nanomachines to achieve rapid and contactless motion. For example, Kopperger et al. created a self-assembled DNA nanorobotic arm that could be positioned on a DNA origami platform by externally applied electric fields, enabling millisecond switching, controlled cargo transport, and force-induced duplex dissociation [86] (Figure 3d). More recently, electric actuation was further developed into a torsion-spring-based DNA nanorobotic system, in which the molecular joint stored mechanical energy under rotating electric fields and released it in a controlled manner through latching and triggering. This work further demonstrated that electric-field responsiveness can be used not only for rapid actuation, but also for programmable mechanical energy storage and release in DNA nanodevices [87].

Electric fields allow rapid, contactless actuation and programmable mechanical energy storage or release in DNA nanodevices. They offer millisecond-scale response and precise control in vitro. Limitations include challenges in vivo due to tissue conductivity, risk of unintended electrochemical effects, and the need for specialized conductive environments.

##### Other Novel Responsive Strategies

Recently, ultrasound-responsive systems have attracted increasing attention as promising platforms for remotely regulating dynamic DNA nanomachines, particularly for biomedical applications requiring non-invasive intervention in deep tissues. Ultrasound exhibits superior tissue penetration capability and can be precisely focused within deep biological regions with minimal attenuation, thereby enabling spatiotemporally controlled activation in vivo [88]. Generally, ultrasound-triggered regulation relies on acoustic cavitation, localized mechanical stress, sonoporation effects, or ultrasound-induced destabilization of responsive carriers, which subsequently induce conformational transformation or therapeutic cargo release [89]. Importantly, ultrasound stimulation can transiently enhance membrane permeability and improve tissue diffusion, thereby facilitating intracellular uptake and drug penetration efficiency. Such characteristics are particularly advantageous for overcoming physiological barriers, including dense tumor matrices and the blood–brain barrier, making ultrasound-responsive systems especially suitable for deep-tissue drug delivery applications [90]. For example, a transformable ultrasound-triggered DNA nanomedicine was recently developed to enhance cascade drug delivery and improve antitumor efficacy through deeper tumor penetration and controlled therapeutic activation [91]. Benefiting from excellent biosafety and mature clinical imaging-guided technology, ultrasound-responsive DNA nanodevices hold great clinical translation potential for deep-seated tumor precise therapy.

Photothermal-responsive systems utilize photothermal agents to convert absorbed light energy into localized heat, thereby inducing structural deformation, dehybridization, or cargo release in DNA nanomachines [92]. Unlike direct photoresponsive regulation based on molecular photoswitches, photothermal strategies mainly depend on thermal transduction generated by nanomaterials such as gold nanoparticles, carbon nanomaterials, and semiconductor nanostructures. Upon near-infrared (NIR) irradiation, localized heating can destabilize DNA duplexes or alter hybridization equilibria, thus enabling remotely controlled actuation and therapeutic release. For instance, Yang et al. developed a biomimetic retractable DNA nanocarrier with sensitive photothermal responsivity for efficient drug delivery and enhanced photothermal therapy, demonstrating improved tumor therapeutic performance under NIR stimulation [93].

Radiation-responsive systems exploit the direct and indirect effects of ionizing radiation (e.g., X-rays, γ-rays) or low-energy electrons to generate strand breaks and nucleobase lesions within DNA nanostructures, thereby triggering programmed dehybridization, conformational collapse, or complete disassembly of the nanomachines. For instance, Sala et al. systematically investigated the stability of DNA origami triangles under X-ray irradiation and demonstrated a dose-dependent loss of structural integrity, with folded origami exhibiting enhanced resistance compared to single-stranded DNA, yet still undergoing controlled fragmentation once a critical damage threshold is exceeded [94]. In a complementary approach, Perry et al. constructed condensed DNA aggregates integrated with gold nanostars; upon irradiation, the gold nanostars amplify secondary electron and free-radical yields, accelerating local DNA damage and inducing rapid aggregate disassembly, thus realizing radiation-triggered dynamic structural change and cargo release [95]. These radiation-sensitive structural transitions offer a promising avenue for constructing smart nanocarriers that deploy therapeutic payloads specifically in the radiation field of a tumor, combining spatial precision with dose-activated release.

In summary, these stimulus-responsive strategies exhibit distinct characteristics in terms of triggering conditions, reversibility, response kinetics, and biocompatibility. To provide a more intuitive and comprehensive overview, a comparative analysis of common stimulus-responsive strategies for dynamic DNA nanomachines is systematically summarized and presented in Table 1.

## 3. Biomedical Applications of Dynamic Nanomachines

The integration of stimulus-responsive modules endows DNA nanomachines with the ability to dynamically reconfigure their structures in response to specific biochemical or physicochemical signals, thereby converting molecular recognition events into programmable functional outputs. This dynamic behavior establishes a direct link between structural transformation and biomedical function, enabling DNA nanomachines to actively regulate signal generation, molecular accessibility, cargo exposure, and therapeutic activation in complex biological environments.

The dynamic and programmable nature of these systems has made biosensing and controlled drug delivery two of the most intensively explored biomedical applications of DNA nanomachines. In biosensing, stimulus-responsive structural transitions can transform specific biomolecular recognition events into amplified and detectable signals with high sensitivity and selectivity. More importantly, the recognition mechanisms can also function as triggers for cargo release, thereby establishing a close functional connection between biosensing and drug delivery. Through such integrated “sense-and-respond” behaviors, dynamic DNA nanomachines enable precise spatiotemporal regulation of therapeutic processes while simultaneously improving diagnostic accuracy and treatment specificity. The following sections will systematically elaborate on the research progress of these dynamic DNA nanomachines in the biomedical field, supported by specific case studies, aiming to provide a reference for technological innovation and biomedical applications in this area.

### 3.1. Biosensing

Dynamic DNA nanomachines are well suited for biosensing because stimulus-induced conformational switching, structural assembly/disassembly, and programmable actuation can directly translate target recognition events into readable optical, electrical, or structural signals. These dynamic processes not only improve sensing sensitivity and specificity but also enable advanced functions such as signal amplification, logic-gated analysis, multiplexed detection, and activatable bioimaging. The following section will systematically elaborate on biosensing strategies based on dynamic DNA nanomachines and their research progress in the fields of detection and imaging.

#### 3.1.1. Sensitive Detection

**Figure 4:**
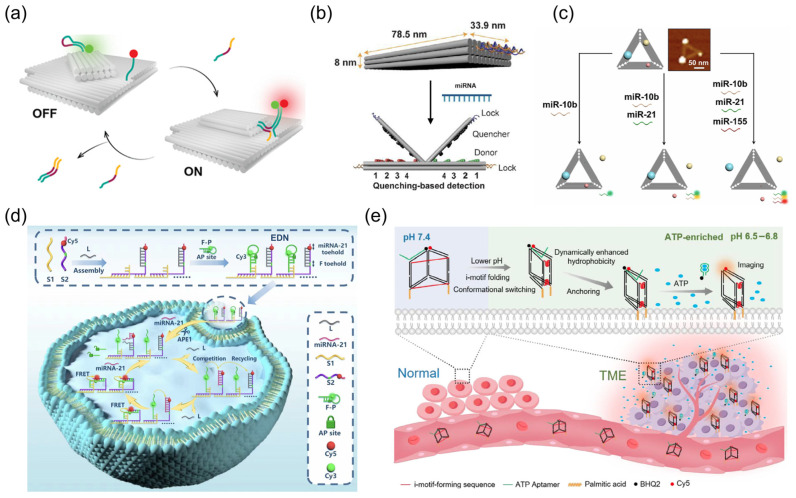
Applications of dynamic DNA nanomachines in biosensing. (**a**) Reconfigurable rotating DNA origami nanodevice for renewable single-molecule nucleic acid detection. Reprinted with permission from ref. [96]. (**b**) Dynamic DNA origami book biosensor for microRNA detection based on target-induced structural opening and quenching-based optical readout. Reprinted with permission from ref. [97]. (**c**) Triangular DNA origami nanosheet carrying three mutually orthogonal DNAzyme walker modules for multiplexed detection. Reprinted with permission from ref. [99]. (**d**) Endogenous cue-responsive DNA nanomachine for intracellular imaging based on programmable activation and fluorescence signal generation. Reprinted with permission from ref. [105]. (**e**) Conformationally switchable hydrophobic tag-conjugated DNA nanoprobes for ATP imaging in the tumor microenvironment. Reprinted with permission from ref. [106].

A key advantage of dynamic DNA nanomachines in sensitive detection lies in the ability of target recognition events to directly drive structural reconfiguration and transduce them into readable signal outputs. This feature not only ensures high sensitivity of the detection method, but also simultaneously endows it with reversibility, renewability, and multiplexed analysis. Nucleic acid detection represents an important direction in sensitive detection. At the single-target level, Tsang et al. developed a reconfigurable rotating DNA origami nanodevice for single-molecule nucleic acid detection. Upon binding of the target strand, the device underwent a defined conformational transition that generated a detectable signal. The nanodevice could then be reset to its initial state by a displacement strand, allowing continuous and renewable detection [96] (Figure 4a). Domljanovic et al. developed a dynamic DNA origami book biosensor for single and dual detection of cancer-associated nucleic acids. In this system, target-induced rotation of the top layer produced either FRET changes or fluorescence dequenching as optical outputs [97] (Figure 4b). More recently, the same platform was further optimized for direct microRNA detection in complex biological fluids. By modifying the lock strands and introducing a polymer additive, the biosensor achieved low-picomolar detection of miR-21 in serum, plasma, and clinical plasma samples without enzymatic amplification, while also supporting simultaneous analysis of miR-21 and miR-7a in clinical specimens [98]. Dynamic DNA nanomachines can also support more complex analytical tasks, such as multiplexed profiling with combined optical and structural readout. In this regard, Huang et al. integrated three mutually orthogonal DNAzyme walker modules onto a triangular DNA origami nanosheet (Figure 4c). Different microRNA targets selectively activated their corresponding walkers, producing three-color fluorescence signals while simultaneously inducing the detachment of gold nanoparticles from predefined positions. As a result, target-dependent nanoscale pattern changes could be directly verified by atomic force microscopy (AFM), enabling multiplexed microRNA analysis with dual signal and structural readout [99].

Moreover, dynamic DNA nanomachines have also been adapted for the recognition of proteins and ions. For protein sensing, Engelen et al. constructed an antigen-responsive DNA origami logic-gated system based on antigen–antibody competitive recognition, in which target binding triggered shell disassembly and generated a detection signal, highlighting the utility of dynamic structural reconfiguration in protein analysis [19]. In ion detection, metal ions can interact selectively with specific bases or DNAzyme modules to induce local coordination, strand cleavage, or conformational transitions, thereby generating readable structural responses. For example, Pb^2+^ can activate DNAzyme-mediated cleavage and induce nanostructure disassembly or local domain opening, thereby producing a readable response [100]. In contrast, Hg^2+^ is commonly recognized through the formation of T-Hg^2+^-T base pairs, which stabilize specific DNA conformations and enable ion-triggered structural switching for selective mercury sensing [101]. Similarly, Ag^+^ can be detected through C-Ag^+^-C coordination, which reconstructs mismatched DNA domains and generates optical or structural readout [102]. Therefore, these examples demonstrate that dynamic DNA nanomachines offer high sensitivity and versatility in biomolecular detection, supporting applications from single-target analysis to multiplexed detection in complex samples.

Overall, leveraging their target-driven structural reconfiguration mechanism, dynamic DNA nanomachines exhibit exceptional sensitivity and versatility across a broad range of biomolecular detection applications, providing a powerful technological platform for achieving precise and intelligent diagnosis in complex samples.

#### 3.1.2. Bioimaging

Bioimaging utilizes functional probes or nanodevices to visually monitor molecular events, structural changes, and microenvironmental characteristics within living organisms or cells. Dynamic DNA nanomachines leverage their dynamic properties to convert intracellular recognition events into activatable imaging signals. Specifically, stimulation by target biomolecules or local microenvironmental cues induces structural transitions, thereby generating signals only under specific biological conditions and enabling intelligent biological imaging. For example, Li et al. developed an endogenous microRNA-activated DNA nanomachine for concurrent intracellular microRNA imaging and activatable gene silencing [103]. This work showed that dynamic DNA systems could integrate molecular imaging with downstream function. Han et al. further introduced a pH-stimulated self-locked DNA nanostructure. The locked design reduced premature activation and enabled more reliable simultaneous detection and imaging of dual microRNAs in cancer cells [104]. Zhang et al. enhanced specificity even further by incorporating endogenous AND logic, whereby signal generation required multiple intracellular cues rather than a single trigger [105] (Figure 4d).

Furthermore, dynamic DNA nanomachines have been widely applied in the visualization of local chemical microenvironments and their associated metabolites or ions. For example, Li et al. developed conformationally switchable hydrophobic tag-conjugated DNA nanoprobes for ATP imaging in the tumor microenvironment. Under weakly acidic conditions, folding of the i-motif brought the two palmitic acid tags into closer proximity, which enhanced membrane anchoring and retained the probe at the target site. This pH-induced structural preorganization enabled selective activation of the nanoprobe for ATP imaging in vivo [106] (Figure 4e). Cui et al. developed an acid-switchable DNAzyme nanodevice in which a pH-triggered structural transition restored the catalytic activity of the DNAzyme in acidic intracellular compartments. Once activated, the DNAzyme converted local metal-ion recognition into signal generation, enabling simultaneous imaging of Zn^2+^ and Pb^2+^ in living cells [107]. This work shows that local chemical environments can also be translated into selective imaging outputs through dynamic structural regulation. As research advances further, dynamic DNA nanomachines are evolving to integrate endogenous biomarkers with exogenous environmental stimuli, thereby achieving more precise spatiotemporal control. Ren et al. reported a near-infrared (NIR) photoswitched nanomachine powered by an endogenous trigger for activatable intracellular microRNA imaging [108]. This concept was further extended to subcellular microenvironment imaging by Gao et al. They constructed a lysosome-targeted NIR/pH dual-activatable DNA nanomachine that integrated a pH-responsive triplex switch with near-infrared-triggered hybridization chain reaction amplification. As a result, lysosomal pH fluctuations could be visualized with spatiotemporal control [109]. More sophisticated regulation was demonstrated by Liu et al. They developed a DNA nanodevice programmatically gated by reductase and light for mitochondrial ATP imaging. In this system, hypoxia-associated reductase first triggered probe release, and subsequent light irradiation activated the imaging module [110]. Xu et al. designed a protein corona-armored DNA nanomachine for multitype biomarker imaging. A UV-responsive protein shell protected the nanomachine during delivery and regulated its intracellular fate. After internalization, light-triggered shell dissociation enabled on-demand activation for spatiotemporally controlled imaging of microRNA and ATP in living cells [111].

### 3.2. Drug Delivery

The high specificity and sensitivity of DNA nanomachine-based biosensing platforms enable real-time monitoring of disease-associated biomarkers, which can in turn serve as triggering signals for on-demand drug release from DNA nanomachine-based delivery systems. Furthermore, many molecular recognition and signal transduction mechanisms originally developed for biosensing have been directly repurposed as regulatory modules for controlled drug release.

Compared with conventional smart nanocarrier systems such as polymeric nanoparticles and peptide-based materials, dynamic DNA nanomachines offer unique advantages in programmability, structural precision, and controllable dynamic regulation owing to the predictable Watson–Crick base-pairing properties of nucleic acids [112]. In particular, their ability to couple molecular recognition with controllable conformational transformation enables more precise regulation of therapeutic activation and cargo release. This key advantage lies in converting in vivo biological signals into precise structural transformations, thereby allowing accurate control over drug loading, transport, and release, and ultimately facilitating targeted and controllable drug release specifically at lesion sites [113,114]. These nanomachines mainly follow two release modes: in one mode, structural opening controls cargo exposure, allowing therapeutic agents to remain shielded during circulation and selectively exposed at target sites; in the other mode, cargo release is induced by carrier destabilization or structural disassembly, which promotes rapid intracellular unloading and enhances delivery efficiency. Although the two strategies differ in their release mechanisms, both can achieve spatiotemporally controlled cargo activation and reduce premature leakage and nonspecific exposure, which is particularly important for minimizing off-target effects during delivery.

#### 3.2.1. Gate-Controlled Mechanisms

**Figure 5:**
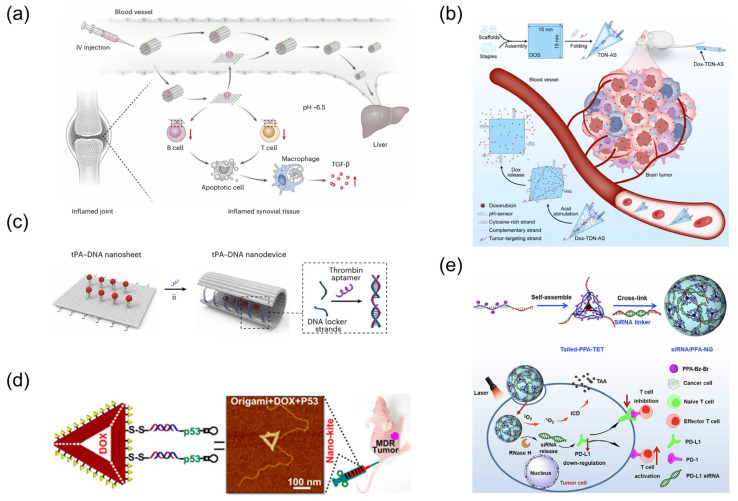
Applications of dynamic DNA nanomachines in drug delivery. (**a**) pH-responsive DNA origami nanodevice for therapeutic activation in inflamed synovial tissue through exposure of a hexagonally patterned CD95 ligand array. Reprinted with permission from ref. [116]. (**b**) Allosteric DNA nanomachines for targeted glioma therapy, featuring blood–brain barrier traversal and acid-triggered doxorubicin release. Reprinted with permission from ref. [117]. (**c**) Threshold-responsive nanodevice based on a thrombin aptamer. Reprinted with permission from ref. [120]. (**d**) DNA nanostructure-based nanocarrier for the co-delivery of a linear p53 gene cassette and doxorubicin. Reprinted with permission from ref. [123]. (**e**) Nucleic acid nanogel for the co-delivery of pheophorbide A and programmed death-ligand 1 (PD-L1) small interfering RNA (siRNA) for synergistic cancer photoimmunotherapy. Reprinted with permission from ref. [128].

The gated release strategy represents one of the pivotal design principles for intelligent drug-delivering DNA nanomachines [115]. A representative example is the reconfigurable DNA origami nanocapsule, which uses programmable pH latches to reversibly switch between open and closed states, thereby enabling cargo loading, encapsulation, and controlled exposure [62]. Li et al. developed a pH-responsive DNA origami nanodevice in which i-motif-based fasteners enabled reversible switching between closed and open states. The device remained closed under physiological conditions, but opened in the weakly acidic microenvironment of inflamed synovial tissue to expose a hexagonally patterned CD95 ligand array, thereby triggering localized death signaling in activated immune cells [116] (Figure 5a). Similarly, Liu et al. developed an allosteric DNA nanomachine for targeted glioma therapy, in which cytosine-rich pH-responsive modules functioned as structural gating elements. These modules maintained the nanomachines in a compact, transport-favorable state under physiological conditions, but underwent protonation-induced reconfiguration in the acidic tumor microenvironment, thereby switching the carrier to an open state and enabling localized release of doxorubicin [117] (Figure 5b). In addition, dynamic DNA nanodevices have also been explored for programmable cancer vaccination. For example, Liu et al. developed a pH-responsive DNA nanodevice vaccine by integrating antigen peptides and molecular adjuvants within a tubular DNA nanostructure [118]. The incorporation of acid-responsive DNA locking strands enabled the nanodevice to remain structurally closed under physiological conditions while undergoing lysosomal activation after cellular internalization, thereby exposing immunostimulatory cargos in antigen-presenting cells. This stimulus-triggered structural transformation significantly enhanced T-cell activation and antitumor immune responses, demonstrating the potential of dynamic DNA nanotechnology for controllable cancer vaccination.

Beyond pH-responsive gating, pathological molecular and enzymatic cues can also be harnessed to regulate carrier permeability or therapeutic exposure with high specificity. Xu et al. constructed dual-chain-locked DNA origami nanocages, in which ATP- or microRNA-21-responsive lock strands regulated cage permeability and enabled on-demand release of Cas9 ribonucleoprotein in tumor cells [119]. Yin et al. developed a threshold-responsive nanodevice based on a thrombin aptamer and interlocked DNA triplexes, in which tissue plasminogen activator (tPA) was exposed only when the thrombin concentration reached a predefined level, thereby enabling precise thrombolysis at thrombus sites [120] (Figure 5c). Zhang et al. reported an inflammation-specific DNA origami nanodevice for ulcerative colitis treatment, in which an apurinic/apyrimidinic endonuclease 1 (APE1)-responsive locking module enabled selective exposure of therapeutic small interfering RNAs in inflamed cells [121]. In addition, gate-controlled dynamic DNA nanomachines also show great potential for gene-editing delivery, where precise spatiotemporal regulation is essential to balance delivery efficiency and off-target effects. By maintaining gene-editing cargos in a protected state during circulation and selectively activating their release in diseased cells, these systems can minimize premature leakage while enhancing intracellular delivery efficiency. For example, Tang et al. developed a DNA origami-based Clustered regularly interspaced short palindromic repeats (CRISPR) associated protein 9 (Cas9) delivery platform in which single-guide RNA/Cas9 complexes were efficiently loaded onto protospacer adjacent motif-rich DNA origami and subsequently locked within a disulfide-crosslinked nanostructure. After targeted cellular uptake and hyaluronic acid peptide-mediated endosomal escape, the nanostructure underwent GSH-triggered opening in tumor cells to release the gene-editing machinery, thereby reducing premature leakage and improving intracellular delivery and gene-editing efficiency in vivo [122].

#### 3.2.2. Structural Disassembly-Enabled Strategies

Complementary to gate-controlled exposure, dynamic DNA nanomachines can also achieve controlled release through structure destabilization, cleavage, dehybridization, or matrix disassembly. These DNA nanomachines typically achieve structural relaxation or dissociation based on either in vivo biomolecule-driven mechanisms or exogenously applied stimuli in vitro, thereby releasing the encapsulated drugs. For example, Liu et al. developed a DNA nanostructure-based nanocarrier for the co-delivery of a linear p53 gene cassette and doxorubicin. In this system, reducible capture strands enabled glutathione-triggered cleavage and release of the gene cargo [123] (Figure 5d). Wang et al. further reported a cell-specific aptamer-modified DNA nanostructure for non-small cell lung cancer therapy. Following cellular internalization, lysosomal degradation broke down the DNA carrier, thereby inducing structural disassembly and intracellular release of doxorubicin [124]. Deng et al. developed uracil-modified DNA nanotubes that respond to the base excision repair enzymes uracil-DNA glycosylase (UDG) and apurinic/apyrimidinic endonuclease 1 (APE1), thereby enabling selective doxorubicin release in cancer cells [125]. In addition to endogenous triggers, externally induced dehybridization can also be used for remote control of release. For example, Pongkulapa et al. developed a near-infrared (NIR)-controlled delivery system based on photoswitchable arylazopyrazole (AAP)-containing DNA strands and upconversion nanoparticles. Near-infrared irradiation triggered DNA duplex dissociation and enabled on-demand release of curcumin. The release stopped when irradiation was removed and resumed upon re-irradiation [126]. Li et al. also developed a DNA origami nanovehicle that enabled near-infrared-triggered, acidic endosomal/lysosomal environment-assisted release of doxorubicin for synergistic chemo-phototherapy [127].

In addition, by integrating tumor-microenvironment-responsive modules with immunoregulatory functions, dynamic DNA nanomachines enable localized activation of antitumor immune responses while minimizing systemic immune-related adverse events. For example, Guo et al. constructed a nucleic acid nanogel by crosslinking photosensitizer-bearing DNA tetrahedral frameworks with programmed death-ligand 1 (PD-L1) siRNA linkers, thereby integrating photodynamic therapy with stimulus-responsive gene release. After cellular internalization, laser irradiation initiated photodynamic therapy, while RNase H-mediated cleavage of the DNA/RNA duplex linkers disrupted the nanogel network and released functional siRNA for PD-L1 silencing [128] (Figure 5e). Wu et al. further reported a mucin-1 and azoreductase dual-responsive DNA tetrahedral nanoprobe for imaging-guided photodynamic immunotherapy [129]. The multi-responsive nanodevice achieved enhanced therapeutic specificity within the hypoxic tumor microenvironment while reducing off-target immune activation.

## 4. Summary and Outlook

### 4.1. Summary

The development and integration of stimulus-responsive modules have driven the evolution of DNA nanostructures into dynamic DNA nanomachines, thereby conferring unique advantages in the biomedical field. This paper provides a systematic overview of response design strategies for dynamic DNA nanomachines and the latest advancements in their applications in biosensing and drug delivery. First, it focuses on the development and implementation of dynamic regulation mechanisms, such as molecular-driven mechanisms and environmental stimulation methods. Notably, response modules enabled by physiologically relevant signals, which are tailored to the demands of the in vivo microenvironment, demonstrate exceptional potential for DNA nanomachines in precision diagnostics and controlled drug release. In summary, the intelligent development of dynamic DNA nanomachines has enabled them to emerge as promising tools in a broader range of biomedical applications.

### 4.2. Outlook

Despite the rapid progress of dynamic DNA nanomachines in biosensing and precision therapeutics, several critical barriers still hinder their clinical translation. One of the primary challenges lies in the large-scale synthesis and manufacturing of structurally complex DNA nanostructures. In particular, the structural heterogeneity of dynamic DNA nanomachines poses significant difficulties for quality control and standardized characterization, especially for systems involving multiple responsive modules or complex conformational transitions. With the continued integration of nucleic acid nanotechnology, artificial intelligence (AI), and biomedical engineering, these key bottlenecks are expected to be gradually addressed. Specifically, AI-assisted approaches can facilitate the optimization of DNA sequences, structures, and responsive modules [130], thereby improving recognition accuracy while preserving structural stability for long-term storage and precise responsiveness. Recent breakthroughs in machine learning have enabled higher-accuracy prediction of DNA reaction kinetics, allowing researchers to efficiently explore relationships among sequence, structure, and function. Transformer-based models, such as DNA-BERT, have been applied to predict strand displacement rate constants directly from sequence features, thereby reducing the need for extensive simulations and experimental optimization during system design [131]. Beyond kinetic prediction, machine-learning approaches are increasingly being used to correlate DNA sequences with structural and functional properties, providing predictive insights into the stability and folding behavior of complex nucleic acid architectures [132]. In the field of DNA logic circuits, emerging computational design frameworks aim to automate the translation of computational specifications into implementable strand-displacement networks. Although fully autonomous compilers are still under development, recent conceptual advances have highlighted the considerable potential of AI-assisted sequence generation and kinetic annotation for large-scale circuit assembly [133].

Another major challenge is that the complexity of the in vivo environment can severely compromise both the structural stability and response accuracy of dynamic DNA nanomachines. Although precise regulation can often be achieved under in vitro conditions, their integrity and functionality are frequently impaired in physiological environments [134,135]. Moreover, in biological systems involving multiple coexisting signals, different response modalities may interfere with one another, thereby reducing response specificity and limiting adaptability to dynamic physiological processes. These limitations ultimately affect both diagnostic reliability and therapeutic precision [136]. To address these challenges, rational surface-engineering strategies, including polymer modification, biomimetic camouflage, and stimuli-adaptive structural stabilization, may improve in vivo stability and prolong systemic circulation. Furthermore, hierarchical regulation, where endogenous cues first define the pathological target region and external stimulation then enables precise temporal activation, can reduce off-target activation and improve operational stability in complex physiological environments.

Systematic biosafety evaluation also remains a prerequisite for clinical translation. Although DNA materials are generally considered biocompatible, chemically modified nucleic acids, artificial response modules, and long-term in vivo accumulation may induce unexpected immunogenicity, inflammatory responses, or organ toxicity. At present, comprehensive studies regarding the long-term safety, biodegradation pathways, and immunological effects of dynamic DNA nanomachines are still limited. Establishing standardized evaluation criteria for toxicity, immunogenicity, and biodegradability will therefore be crucial for accelerating preclinical development and regulatory approval. Future efforts should therefore promote interdisciplinary collaboration among nanotechnology researchers, biomedical engineers, clinicians, and regulatory agencies to establish standardized manufacturing specifications, characterization guidelines, and translational evaluation frameworks for dynamic DNA nanomedicines.

## Figures and Tables

**Table 1:** Comparative analysis of different stimulus-responsive strategies for dynamic DNA nanomachines.

Response Strategy	Advantage	Disadvantage
Nucleic acids	Highly programmable and modular, with precise control over complex structural reconfigurations	Susceptible to nonspecific interactions
Enzyme	Highly specific and programmable control	Enzyme activity is sensitive to environmental conditions such as pH and temperature
Small molecules	Autonomous activation	Biomarker heterogeneity
pH	Simple to implement and highly programmable	Slow response kinetics and narrow physiological window
Ions	Reversible	Requiring careful tuning of ion concentrations
Light	Non-invasive, reversible, and High spatiotemporal precision	Limited penetration
Temperature	Reversible and controllable hybridization kinetics	Narrow physiological temperature window and potential thermal damage to surrounding tissues or DNA cargos
Magnetic field	Remote, reversible control and can penetrate complex media	Increases system size and complexity
Electric field	Rapid activation and contactless actuation	Need for specialized conductive environments

## Data Availability

Data are available in a publicly accessible repository.
